# Contemporary Urban Space Philosophy in China Using Lightweight Deep Learning Model-Under Ecological Ethics

**DOI:** 10.1155/2022/8925205

**Published:** 2022-03-18

**Authors:** Haiyan Cheng

**Affiliations:** ^1^Institute of Economic Ethics, Shanghai University of Finance and Economics, Shanghai 200433, China; ^2^Shanghai University of Finance and Economics Zhejiang College, Zhejiang 321000, China

## Abstract

It aims to improve the construction of ecological civilization and promote the common development of urban and ecology. Firstly, contemporary ecological ethics is explored, and its principles and characteristics are summarized. Then, the technique of convolutional neural network (CNN) image in a deep learning model is analyzed. Finally, deep convolutional neural networks (DCNN) are used to analyze and model the spatial characteristics of contemporary cities based on ecological ethics. According to the investigations, rural residential areas are more consistent with ecological ethics than urban residential areas when compared with the ecological characteristics of farmland and forest, and the highest ecological eigenvalues of the two areas are about 8 and 6. In the analysis of urban space, the maximum value of ecological eigenvalues of an airport is 9, and that of a stadium is 8. However, the scope of their construction that is consistent with ecological ethics is very small. Moreover, the eigenvalues of ecological ethics in the urban business circle of casinos are not only very low (the highest values are about 5 and 3), but also consistent with the construction norms of ecological ethics. The work of urban spatial philosophy is optimized based on the adoption of the DCNN model of deep learning in ecological ethics, which not only provides the reference for future ecological urban planning but also contributes to the common development of urban and ecology.

## 1. Introduction

Contemporary society is developing very rapidly, and urban construction is also in full swing. However, when human beings develop, they must consider the development of the Earth because the development of human beings cannot be done without the nurturing of the Earth [1]. In the development of the Earth, the ecological environment is indispensable, and its proportion is the highest. Therefore, the construction of ecological civilization needs to be more relevant in contemporary urban construction; that is, the construction of urban space needs to be more in line with the norms of ecological ethics [2]. In the high-speed period of technological development, deep learning algorithms can be used to extract the structural features of urban space; and analysis of the characteristics of urban space can provide a plan that is more in line with the construction of ecological civilization in future cities [3]. Although the construction of ecological civilization is not perfect in the current urban construction, there are many kinds of research providing technical support for it.

Bian et al. [4] pointed out that the city is a product of the development of social productivity to a certain stage, and it is an artificial ecosystem created by mankind. The population here is densely populated, the productivity is highly developed, a large amount of material and wealth are collected, many materials and energy flow, and lots of waste are discharged into the environment. The urban ecosystem is a natural, economic, and social complex system created by urban residents. It is not a complete and self-stable ecosystem, but a huge open system. Song et al. [5] pointed out that with the rapid development of industrialization and the acceleration of urbanization on a global scale, China's urbanization process has entered a stage of comprehensive development. Wang et al. [6] pointed out that, on the one hand, urbanization has played an important role in promoting the development of society, the economy, transportation, production, technology, and culture, which are covered in the conceptual framework of the urban economic system. On the other hand, the metabolic function of the city itself is having a major impact on the ecological balance of the natural world due to its continuous growth, high concentration of urban population, and the continuous increase in consumption demand. It also causes various adverse effects on the living environment and life quality of human beings. Urban ecological construction and urban economic competitiveness are two important subsystems in the complex system of human and natural development. They interact with and influence each other to jointly promote the healthy, rapid, and harmonious development of the city. In the process of urban ecological construction, the optimization of the urban human settlement environment and the promotion of urban economic competitiveness should promote each other and be harmonious and unified. However, in the traditional energy-consuming production model, urban construction ignores the environmental carrying capacity and promotes the economic and social development of the city at the expense of limited resources and the natural environment on which humans depend. In the wake of the global greenhouse effect and increasingly serious environmental pollution, cities should actively promote ecological construction, pay attention to environmental pollution control issues, continuously optimize environmental processing capabilities, and strive to achieve a virtuous cycle of urban materials, energy, and information. Only by improving and optimizing the living environment through urban ecological construction can human beings attract more advantageous resources to serve the improvement of the city's economic level, thereby enhancing the overall competitiveness of the city. Miao et al. [7], based on eco-philosophical thoughts, guided by the three urban ecology perspectives of life, dynamic, and system perspectives, they followed the three-legged thinking mode of “views, ideas, and practices” to analyze the “false ecology-false ecology” in the construction of ecological space and comprehensively understood the ecological connotation of the construction of “four modernizations” from the perspective of ecology. It is hoped that through the comprehensive promotion of “quality-enhanced greening, seasonal colorization, preservation of preciousness, and diversification of benefits,” the urban green life landmarks with “near-natural forests” as their mainstay can be scientifically built, realizing the ecological suitability of an excellent global city. Esteva et al. [8] pointed out that deep learning is a research field that has attracted much attention in recent years and plays an important role in machine learning. If shallow learning is a wave of machine learning, then deep learning, as a new field of machine learning, will set off another wave of machine learning. Deep learning is established to simulate the hierarchical structure of the human brain to extract features from low-level to high-level externally input data so as to be able to interpret external data. Valueva et al. [9] pointed out that with the advent of the era of big data, deep convolutional neural networks (DCNN) with more hidden layers have a more complex network structure and show more powerful feature learning and feature expression abilities than traditional machine learning methods. The convolutional neural network (CNN) model trained using deep learning algorithms has achieved impressive results in multiple large-scale recognition tasks in computer vision since it was proposed. Therefore, the use of DCNN for image feature extraction is faster and more accurate.

To sum up, the DCNN algorithm based on ecological ethics is used to analyze the ecological philosophy of urban space. Firstly, ecological ethics is explored, and its principles and characteristics are summarized. Then, based on the explanation of the deep learning method, the research method for DCNN is determined. DCNN includes the input layer, convolution layer, pooling layer, full connection layer, and Softmax layer, so the results obtained during the task processing are comprehensive and reliable. Finally, the characteristics of different urban construction spaces are extracted and classified by comparing them with farmland and forest. The research results not only provide suitable techniques and methods for the construction of urban ecological civilization in the future but also contribute to the development of ecological urban.

## 2. Urban Philosophy Based on Ecological Ethics

### 2.1. Basic Theory of Ecological Ethics

**Figure 1:**
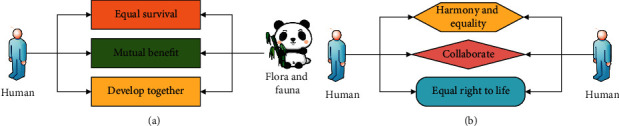
The comprehensive principles of ecological ethics: (a) shows the relationship among people, animals, plants, and nature and (b) shows the relationship between people and people.

Ecological ethics refers to all the ethics adopted by people when dealing with the relationship between themselves, animals, plants, and the ecological environment in the process of survival [10]. It is generally formed through the long-term relationship between human beings and nature, and this ethical relationship contains information on the historical development of the relationship between human beings and nature. In the ecological ethics development information, there are many concepts to promote the common development of humans and the ecology, such as “protecting ecological harmony,” “harmonious coexistence between man and nature,” and “maximizing symbiosis” [11]. In the historical development of China, traditional ecological ethics thought was mainly developed from the Warring States Period [12]. The ecological ethics ideas of Taoism and Confucianism are more prominent. Through the integration and extraction of Taoism and Confucianism, the idea of harmony between man and nature can be obtained, which requires human beings to be unified with nature and live in harmony. In Confucianism, man and nature are a whole, and they coexist and are equal to each other. Therefore, Confucianism points out that human beings need to be in harmony with nature in the process of survival. In the modern ecological ethics system, the concept of harmony between man and nature is composed of equality between man and nature and equality among mankind. The concept of equality between man and nature refers to the treatment of the relationship between man and nature, emphasizing the equality between man and nature. It means that nature and man have the same status in terms of survival, and man and nature have their own specific important values. The concept of equality among mankind refers to the need to achieve equality among people when dealing with relationships in the process of getting along with people. That is, the survival and development of some people should not be a threat to the survival and development of others, and they need to survive and develop equally [13]. The specific principles of ecological ethics are shown in Figure 1.

**Figure 2:**
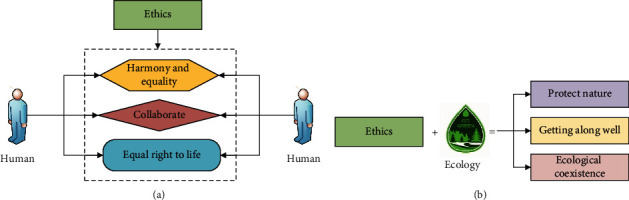
The basic principles of ethics and the principles of ethics combined with ecology: (a) shows the ethical principles and (b) shows the principles of ethics combining with ecology.

As shown in Figure 1, a perfect ecological ethics concept of getting along with nature has been gradually formed in the long-term survival and development process of human beings. The traditional concept of ecological ethics is the basis for the development of the modern concept of ecological ethics. In the concept of modern ecological ethics, the concept of equality between man and nature and the concept of human equality are the main central theories. The analysis of ecological ethics reveals that ethics is a concept that exists in a virtual state, and its main principle is a relational theory [14]. In the long-term human survival process, ethics has gradually transformed into human relations and social order in social survival through the treatment of interpersonal relationships and the relationship between man and nature. Through the combination of ecology and it, it can clearly show a form of harmonious coexistence between man and man and between man and nature, that is, the development model of the unity of human society and the unity of man and natural society. Judgment through this model can be used to express the specific value of people in the process of getting along with people and nature [15].  Figure 2 shows the basic principles of ethics and the specific manifestations of ethics combined with ecology.

**Figure 3:**
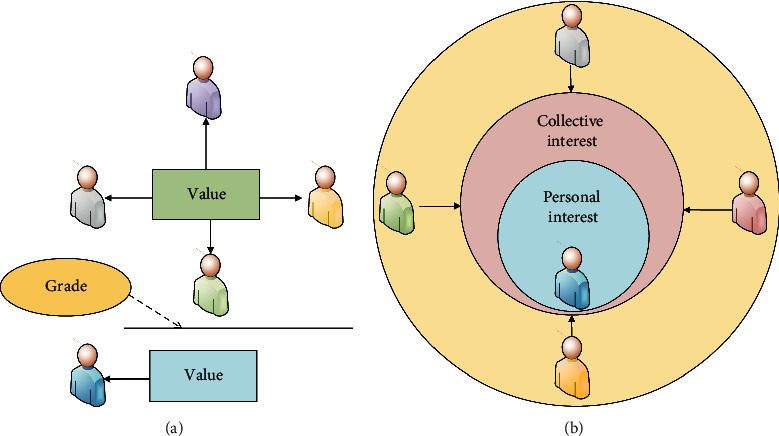
Personal value/benefits and collective value/benefits: (a) shows the personal value and collective value and (b) shows the personal benefit and collective benefit.

As shown in Figure 2, ethics is a manifestation of the relationship between man and man and between man and nature. Through its specific performance, the future development of mankind can be relatively regulated. Then, it can combine ethics with ecology to find that in the process of human survival and development, the ethics of man-to-man and man-to-nature are a basic direction for human long-term survival [16]. Through the combination of ecology and ethics, human beings have gradually established a reasonable concept of ecological ethics for their survival, and the formation of the concept of ecological ethics has many characteristics [17]. First, the concept of ecological ethics indicates that the common value of society is higher than the value of individuals. That is, human beings should always put the value of society first in the process of survival and development. In this way, the concept of ecological ethics requires that individual values always obey the common values of society. When personal interests and common interests of society are dealt with, it has to meet the requirements of social interests first [18].  Figure 3 shows the main principle that social common value is higher than individual value.

**Figure 4:**
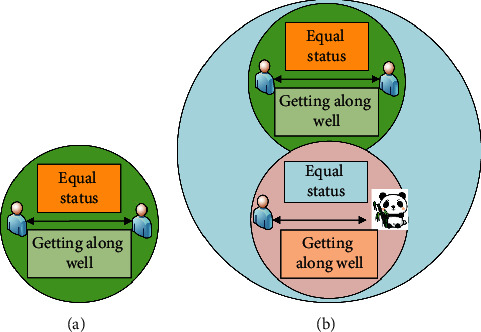
Ethics and ecological ethics: (a) is ethics and (b) is ecological ethics.

As shown in Figure 3, the prerequisite for individuals to realize their self-worth is to first satisfy the realization of common social values, and ecological ethics requires that personal values and interests always obey social values and interests. Second, the concept of ecological ethics adds to the normative content of ethics. Ecological ethics not only regulates the relationship between people and society, but ecological ethics also regulates the relationship between people and nature. Among the added content of getting along with nature, the main manifestation is that people and nature should live in harmony. In the process of survival and development, humans should reasonably handle the relationship between humans and animals and plants. Humans should protect humans, animals, plants, and the ecological environment accordingly, and should not cause damage to animals, plants, and the ecological environment in the process of their own development. Common development requires human beings to put themselves and the ecological environment in an equal position [19].  Figure 4 shows the concrete manifestation of ecological ethics' expansion of ethics.

As shown in Figure 4, the specific performance of ecological ethics provides good ethics for the survival and development of mankind and gives the correct guidance for mankind and nature to live in harmony.

## 3. Space Philosophy Theory

**Figure 5:**
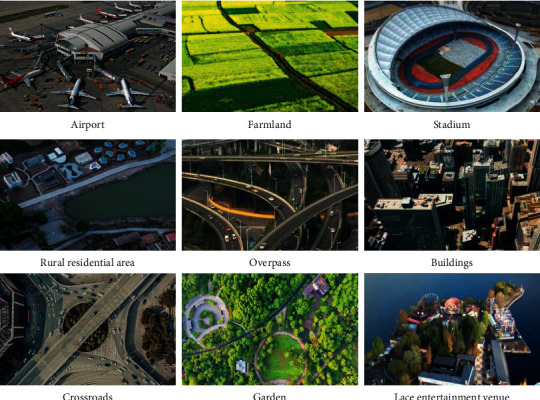
Spatial structure characteristics.

Space theory refers to a theory in which human beings perceive the space in which they are located. Space is not only a practical object produced by humans but also an important subject of human production activities. It is not only an object in human cognition but also a virtual structure in human cognition. The cognition of human existence through space also improves the correct cognition of the universe and society [20]. The space theory provides a correct theoretical framework for mankind to construct a reasonable space structure and offers support for the proper development of mankind. Space theory includes space epistemology, space value theory, and space methodology [21]. In space epistemology, the combination of space and time provides important support for the construction and development of space. In the process of space development, the cognition of time can affect the cognition of space. Therefore, in the epistemology of space, a firm and correct cognition of space is needed to ensure that space can play a correct guiding role in the development of human society [22]. In the theory of space value, the development of space needs to be carried out in the direction of justice, and the development of space needs to play a positive role in the development of mankind. The development of space in the direction of justice is the main channel for its value. Therefore, human beings need to combine the direction of human development when constructing space, construct a reasonable space, and promote human development. In the theory of space value, the basic requirements for building space also include a scientific theoretical system. With the development of society, the scientific theoretical system has become the basic direction of human development. Therefore, the construction of human development space also needs the assistance of scientific theory [23]. In the space methodology, the construction of space not only satisfies human development but also needs to reflect the value of space, which provides support for the reasonable construction of space [24].  Figure 5 shows the main principles of space theory.

As shown in Figure 5, in the process of human development, the support of space theory is needed, and the construction of space needs to be supported by cognition that conforms to its justice, scientific value, and reasonable development theory [25]. Therefore, in the construction of contemporary cities, the space needs to be constructed reasonably to meet the sustainable development of human society.

## 4. Deep Learning Methods

With the development of science and technology, the application of deep learning has become one of the main technical channels for current social development [26]. As a branch of machine learning, deep learning has multiple data processing centers and an abstract computing model that can process data in multiple batches. The deep learning model includes an input layer, a hidden layer, and an output layer. The hidden layer is the most complex level and contains many computing centers [27]. In image processing, DCNN is the earliest deep learning model to be applied. It is also very suitable for multidimensional data processing through local connections, weight sharing, pooling, and multilayer structure [28]. Therefore, the urban space structure is analyzed in this work using the CNN algorithm, and the CNN calculation equation is as follows:

(1) $$\begin{array}{c} f\left(X\right)=\sum_{\mathrm{i=1}}^{L}X_{j}*W_{i}+b_{j}. \end{array}$$

In the above equation, X represents the output values of each layer, *i* represents the layer of CNN, *W* represents the weight matrix of CNN, *b* represents the bias vector of CNN, and *j* represents the layer below the current layer. The following equation shows the calculation of objective function:

(2) $$\begin{array}{c} J\left(W,b\right)=-\frac{1}{m}\overset{m}{\underset{i=1}{\sum }}\left[y^{\left(i\right)}\times \;\log\;h_{W,b}\left(X^{m}\right)+\left(1-y^{\left(i\right)}\right)\times \log\left(1-h_{W,b}\left(X^{m}\right)\right)\right]. \end{array}$$

In the above equation, *m* expresses the number of training samples, *y* expresses the label of the sample, and *h* expresses the loss of layer. DCNN algorithm is used to process contemporary urban spatial images. The contemporary space philosophy is investigated through the analysis of space characteristics [29].

## 5. Network Coding and Blockchain Technology Model Design

**Figure 6:**
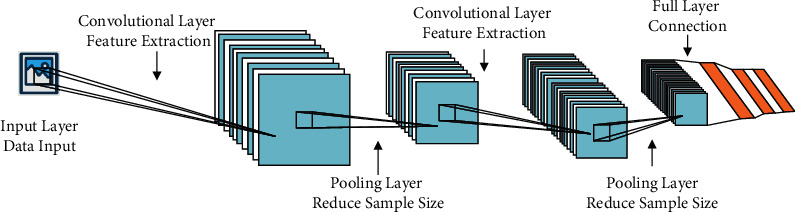
Basic principles of CNN model.

CNN model is adopted to analyze the characteristics of contemporary urban space. Firstly, the basic framework of CNN is built. According to CNN, the geographic features of different places are analyzed. The main technology consists of analyzing the specific eigenvalues of different places through the extraction of image eigenvalues. Subsequently, according to the size of its eigenvalues, the degree of its conformity with ecological ethics is analyzed. Eventually, according to the results of the analysis, a comprehensive analysis of the urban spatial structure is performed, and an ecological urban space is established, thus promoting the development of the city. The calculation of the CNN model involves extracting the eigenvalues of images at each layer and outputting the eigenvalues through the final weight matrix. The CNN model includes a convolution layer, a pooling layer, and an excitation layer. The combination of these levels is used to extract and output images with eigenvalues of different levels [30].  Figure 6 shows the specific calculation principle of CNN.

Figure 6 shows that in the calculation process of the CNN model, the convolutional layer is used to extract the next characteristics of the image. Then, the image characteristics are refined and simplified by the pooling layer so that the eigenvalues are extracted conveniently and quickly. The specific errors of image characteristics analysis are extracted by the inverse calculation method of CNN. The parameters of the image characteristic extraction model is adjusted by the errors. Compared with traditional image analysis technology, it is more comprehensive and reliable [31].

**Figure 7:**
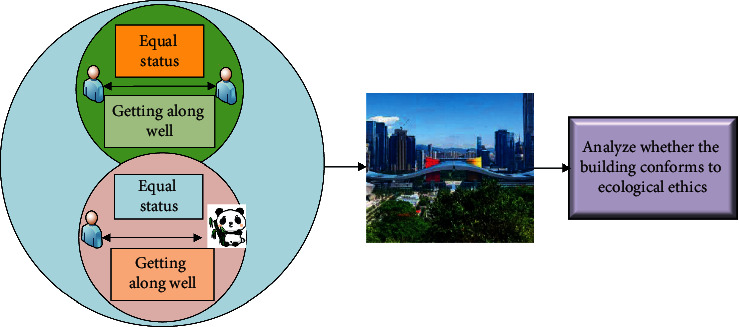
Analysis of urban architecture based on ecological ethics.

The analysis of image characteristics is based on the theory of ecological ethics. It is necessary for the analysis of contemporary urban space characteristics to satisfy the basic ethics of harmonious coexistence between man and nature. Through ecological ethics analysis, both the specific ecological characteristics of contemporary urban space and the specific philosophical principles of contemporary urban spatial structure are analyzed. Compared with the traditional analysis of urban spatial characteristics, the deep learning technology adopted is more comprehensive and reliable, which helps comprehensively analyze urban systems from various aspects and improves the ecological quality of urban structures [32].  Figure 7 shows the specific principles of urban spatial characteristics analysis under ecological ethics.

As shown in Figure 7, ecological ethics is the basic law of human existence. The analysis of contemporary urban space structures based on ecological ethics is to extract the ecological ethics features of urban space; the analysis of philosophical thinking is conductive to analyzing the ecological rationality of contemporary urban space structures so as to provide a specific direction for the development of human ecological civilization.

## 6. Analysis on Urban Architecture Based on Ecological Ethics

### 6.1. Building Structure Analysis

**Figure 8:**
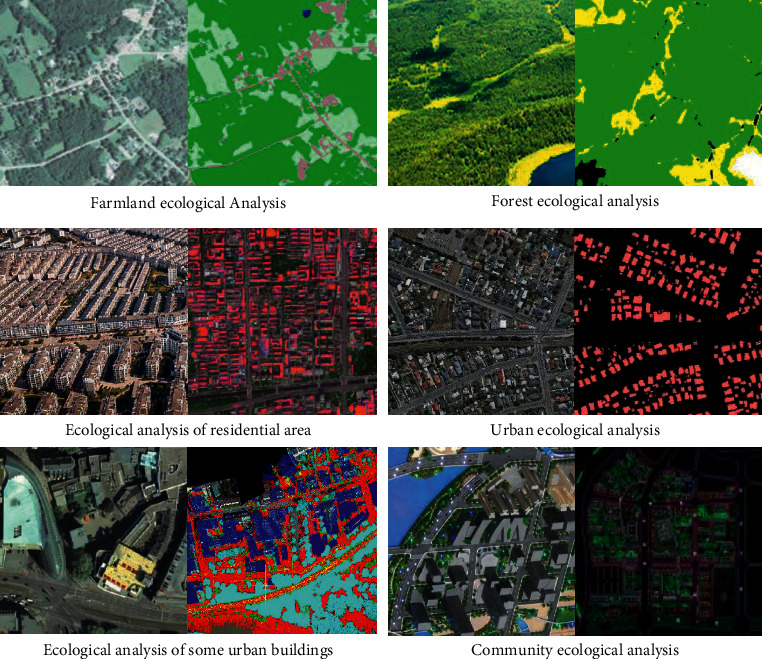
Analysis of the image characteristics of urban buildings.

Deep learning algorithms are adopted to extract the features of urban buildings and analyze the construction features of the buildings, making it possible to explore whether the buildings conform to ecological ethics and analyze their ecological level of buildings. First, it is necessary to extract the features of the building through DCNN and analyze the ecological characteristics of the building through feature extraction and image processing. The image processing results of the building structure are displayed in Figure 8.

As shown in Figure 8, when the image analysis is performed on farmland, forests, and urban architectural spaces, green represents vegetation coverage, and the remaining colors represent other objects that are inconsistent with ecological conditions. In the figure, the more obvious the contrast with green, the lower the ecological level. The ecological level of farmland and forest is the highest, while the ecological level of urban buildings and residential areas is relatively low. The comprehensive reason can be analyzed as that the urban buildings are too dense and the greening work is not perfect.

### 6.2. Urban Comprehensive Ecological Characteristics

**Figure 9:**
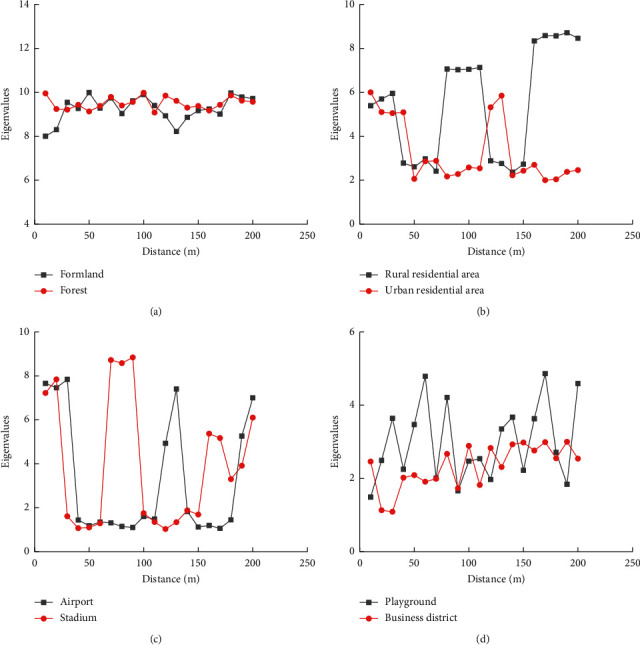
Comparison of urban space ecological characteristics. (a, b, c, and d showed comparisons in different spaces).

To analyze urban space through deep learning algorithms, it is necessary to extract the image features of urban space and classify the feature values based on green to analyze the specific ecological level of urban space.  Figure 9 shows the results of the ecological characteristics analysis of part of the urban space.

In Figure 9, a deep learning algorithm is used to process urban spatial images. Through the extraction of the eigenvalues, the ecological characteristics of different urban spaces are compared. Farmland and forests are used as a comparison due to their spaces with high ecological characteristics. The highest eigenvalue of the two is 10, and the overall value remains between 9 and 10, which indicates that they are consistent with ecological ethics. After comparison, the ecological characteristic values of rural and urban houses are relatively low. The highest eigenvalues of the two are about 8 and 6, and the eigenvalues of the rural area remain 2–9, and those of the urban area remain 2–6. Hence, the rural residence is more consistent with the standard of ecological ethics than the urban residence. The extraction and analysis of urban spatial characteristics show that the maximum ecological eigenvalues of airports and stadiums is high. The maximum of airport eigenvalues is around 9, and that of stadium eigenvalues is around 8. The eigenvalue of the airport remains 1–8, and that of the stadium remains 1–9, so the overall ecological eigenvalues of the two are very low. Consequently, airports and stadiums have relatively little ecological space. Furthermore, the eigenvalue and ecological scope of an amusement park and an urban business district are very small. The highest eigenvalue of an amusement park is about 5, and that of a business circle is about 3. The eigenvalue of amusement parks remains between 1 and 5, and that of urban business areas between 1 and 3.

## 7. Conclusion

Based on ecological ethics, deep learning methods are used to analyze the urban spatial structure. Urban spatial structure is extracted and classified according to the characteristics of ecological ethics. The DCNN method provides the main technical support for the analysis of urban spatial structure. The DCNN method is used for comprehensive analysis of urban structure in many aspects and levels, which improves the final effect of ecological analysis of urban spatial structure. Compared with farmland and forest land, rural residential areas are more consistent with ecological ethical standards than urban residential areas, which indicates that the construction of rural residential areas is consistent with ecological characteristics. The analysis of other constructions in the city showed that the maximum ecological eigenvalues of the airport and stadium are relatively high, at about 9 and 8. Nevertheless, the continuous scope of maximum ecological eigenvalues is relatively small, so the scope of construction in accordance with ecological ethics is also relatively small. According to the analysis, the maximum values of ecological characteristics for casinos and urban business districts are relatively small, at around 5 and 3. Hence, there are few structures that conform to ecological ethics in construction. Besides, the scope of contemporary urban architectural space conforming to ecological ethical standards is very small. To promote the construction of ecological civilization and urban development, more architectural space needs to be added in the future urban space planning in accordance with ecological ethical standards to promote the construction of ecological civilization and urban development. Although the results of this work provide several advanced technical methods, their practical adoption and feature extraction are not perfect. In the future, the practical adoption and the extraction of urban spatial characteristics will be strengthened, as well as the ecological construction of urban areas.

## Data Availability

The data used to support the findings of this study are included within the article.
